# Crystal structure and Hirshfeld surface analysis of 10-(3-benzyl­thio­phen-2-yl)-5,5-di­fluoro-5*H*-4λ^4^,5λ^4^-di­pyrrolo­[1,2-*c*:2′,1′-*f*][1,3,2]di­aza­borinine

**DOI:** 10.1107/S2056989026003889

**Published:** 2026-04-29

**Authors:** Zlata A. Polianskaia, Artem S. Larionov, Victor N. Khrustalev, Mehmet Akkurt, Gizachew Mulugeta Manahelohe, Narmina A. Guliyeva, Khudayar I. Hasanov

**Affiliations:** a RUDN University, 6 Miklukho-Maklaya St., Moscow 117198, Russian Federation; b Zelinsky Institute of Organic Chemistry of RAS, Leninsky Prospect 47, Moscow 119991, Russian Federation; c Department of Physics, Faculty of Sciences, Erciyes University, 38039 Kayseri, Türkiye; d Department of Chemistry, University of Gondar, PO Box 196, Gondar, Ethiopia; e Department of Chemical Engineering, Baku Engineering University, Khirdalan Hasan Aliyev str. 120, Baku, Absheron AZ0101, Azerbaijan; f Azerbaijan Medical University, Scientific Research Centre (SRC), A. Kasumzade St. 14, AZ 1022, Baku, Azerbaijan; Tokyo University of Science, Japan

**Keywords:** crystal structure, C—H⋯F inter­actions, C—H⋯π inter­actions, Hirshfeld surface analysis

## Abstract

In the title compound, C_20_H_15_BF_2_N_2_S, the twelve-membered ring system is essentially planar (r.m.s. deviation = 0.001 Å). The dihedral angles between the average plane of this ring and the thio­phene and phenyl rings are 58.69 (4) and 61.41 (4)°, respectively. In the crystal, C—H⋯F inter­actions generate *R*^2^_2_(10) and three types of *R*^2^_3_(21) ring motifs around a mol­ecule, resulting in layers parallel to the (101) plane. The mol­ecules further form layers parallel to the (101) plane through C—H⋯π inter­actions. According to a Hirshfeld surface analysis, H⋯H (41.5%), C⋯H/H⋯C (23.5%) and F⋯H/H⋯F (18.2%) inter­actions are the most significant contributors to the crystal packing.

## Abstract 2

In the crystal, the C—H⋯F inter­actions between mol­ecules form layers parallel to the (101) plane. Additionally, the mol­ecules also form layers parallel to the (101) plane through C—H⋯π inter­actions.

## 1. Chemical context

**Figure 1:**
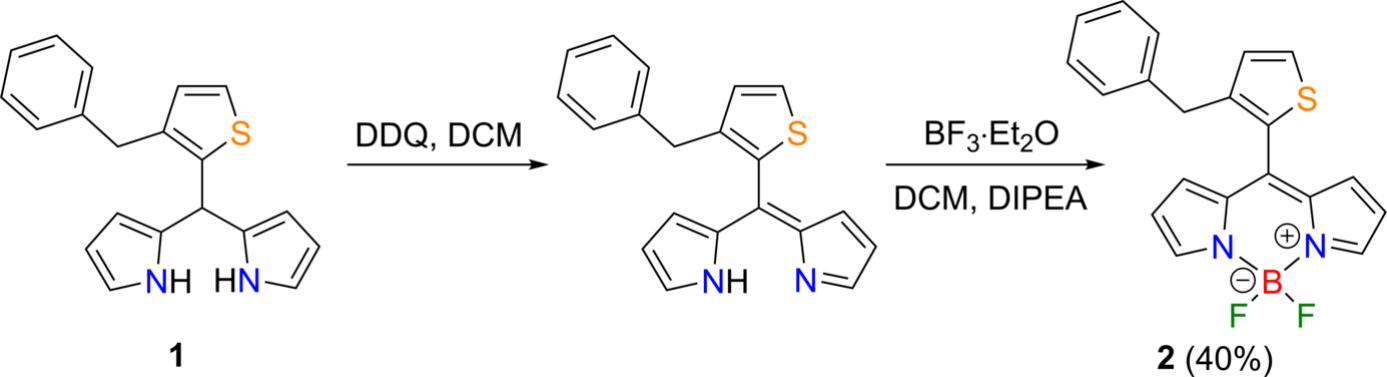
Synthesis of 10-(3-benzyl­thio­phen-2-yl)-5,5-di­fluoro-5*H*-4*λ*^4^,5*λ*^4^-di­pyrrolo­[1,2-*c*:2′,1′-*f*][1,3,2]di­aza­borinine, **2**.

4,4-Di­fluoro-4-bora-3a,4a-di­aza-s-indacene (BODIPY) com­plexes represent one of the most versatile classes of small-mol­ecule fluoro­phores, renowned for their exceptional photophysical properties (Ulrich *et al.*, 2008 ▸; Loudet & Burgess, 2007 ▸). These properties include high molar absorption coefficients, sharp fluorescence emission peaks with high quantum yields, and remarkable chemical and photochemical stability (Boens *et al.*, 2019 ▸; Ni & Wu, 2014 ▸). Consequently, BODIPY derivatives have found extensive applications across diverse fields, functioning as fluorescent sensors for bioimaging, agents for photodynamic therapy, laser dyes, and photocatalysts (Poddar & Misra, 2020 ▸; Martynov & Pakhomov, 2021 ▸; Wang *et al.*, 2023 ▸). A key feature that underpins their versatility is the ability to fine-tune their spectroscopic characteristics through rational structural modification of the dipyrromethene core (Waly *et al.*, 2022 ▸; Lu *et al.*, 2014 ▸). The photophysical behavior of BODIPY is highly sensitive to the nature of the substituent at the *meso*-position (C-8), which plays a critical role in the mol­ecule’s electronic distribution (Ozdemir *et al.*, 2014 ▸; Lincoln *et al.*, 2014 ▸). While the introduction of aryl substituents at this position is well-documented (Spector *et al.*, 2024 ▸), replacing the six-membered ring with five-membered aromatic heterocycles, which can lead to the further modulation the electronic properties, are poorly studied. Studies of *meso*-substituted BODIPY with such heterocycles as furan, thio­phene, pyrrole and seleno­phene were firstly carried out and demonstrated that this substitution results in a more planar conformation between the heterocycle and the dipyrromethene framework, extending π-conjugation and leading to notable bathochromic shifts in absorption and emission spectra, as well as changes in fluorescence quantum yields, compared to their *meso*-aryl counterparts (Kim *et al.*, 2010 ▸; Sharma *et al.*, 2016 ▸). In this work, we describe the synthesis of a new BODIPY derivative bearing a thio­phene moiety at the *meso*-position to explore its impact on the structural, electronic, and photophysical characteristics of the resulting fluoro­phore. In continuation of our studies on five-membered-heterocycle-substituted dipyrrolmethanes, the recently described 3-benzyl-2-[bis­(1*H*-pyrrol-2-yl)meth­yl]thio­phene, **1** (Sadikhova *et al.*, 2024 ▸) was utilized as a key precursor. Oxidation with DDQ in CH_2_Cl_2_ (30 min), followed by neutralization with DIPEA and subsequent treatment with BF_3_·OEt_2_, provided the corresponding BODIPY complex. The target *meso*-thienyl-substituted BODIPY, **2**, was isolated in 40% yield after silica gel column chromatography (Fig. 1 ▸).
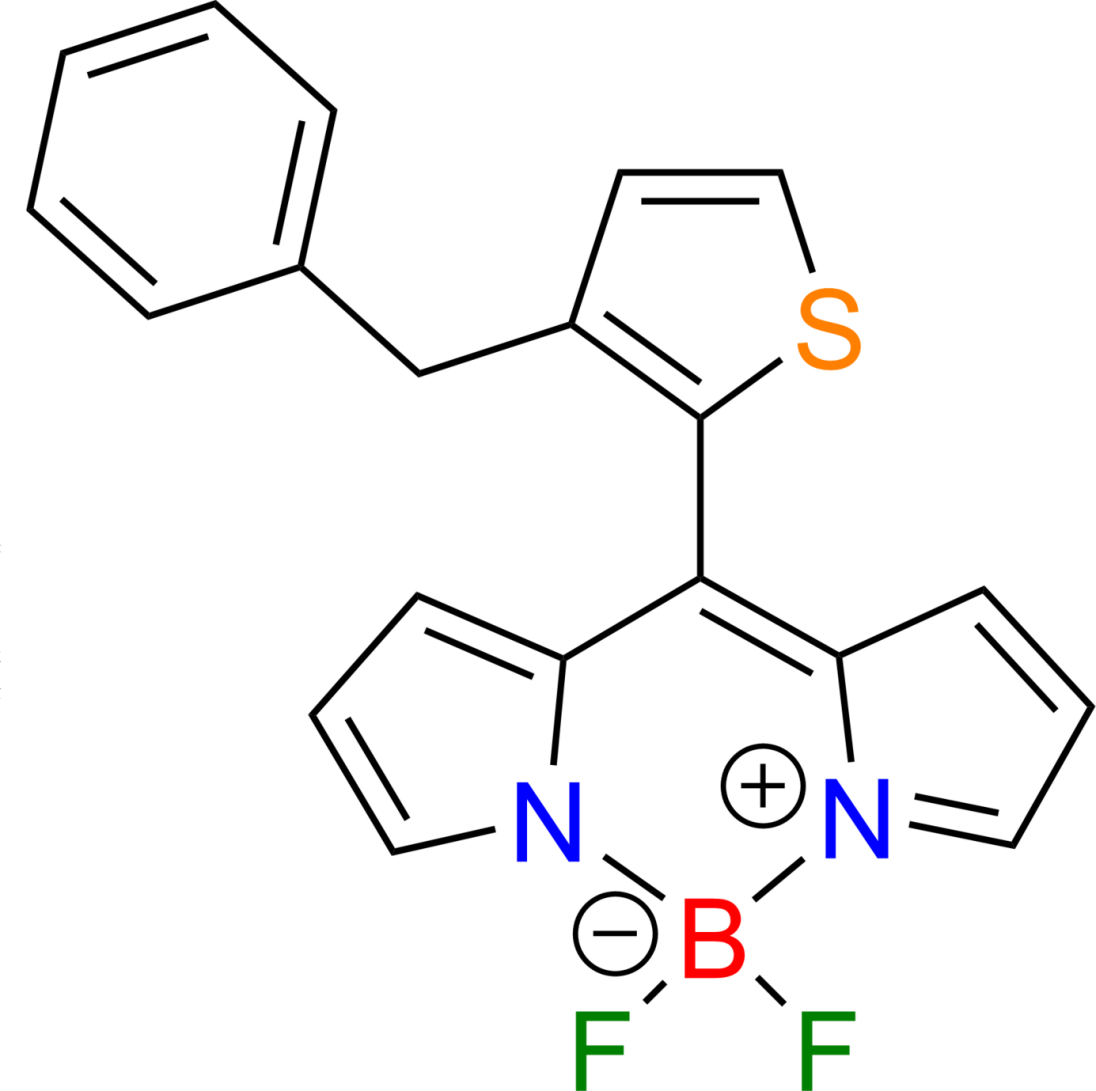


## 2. Structural commentary

**Figure 2:**
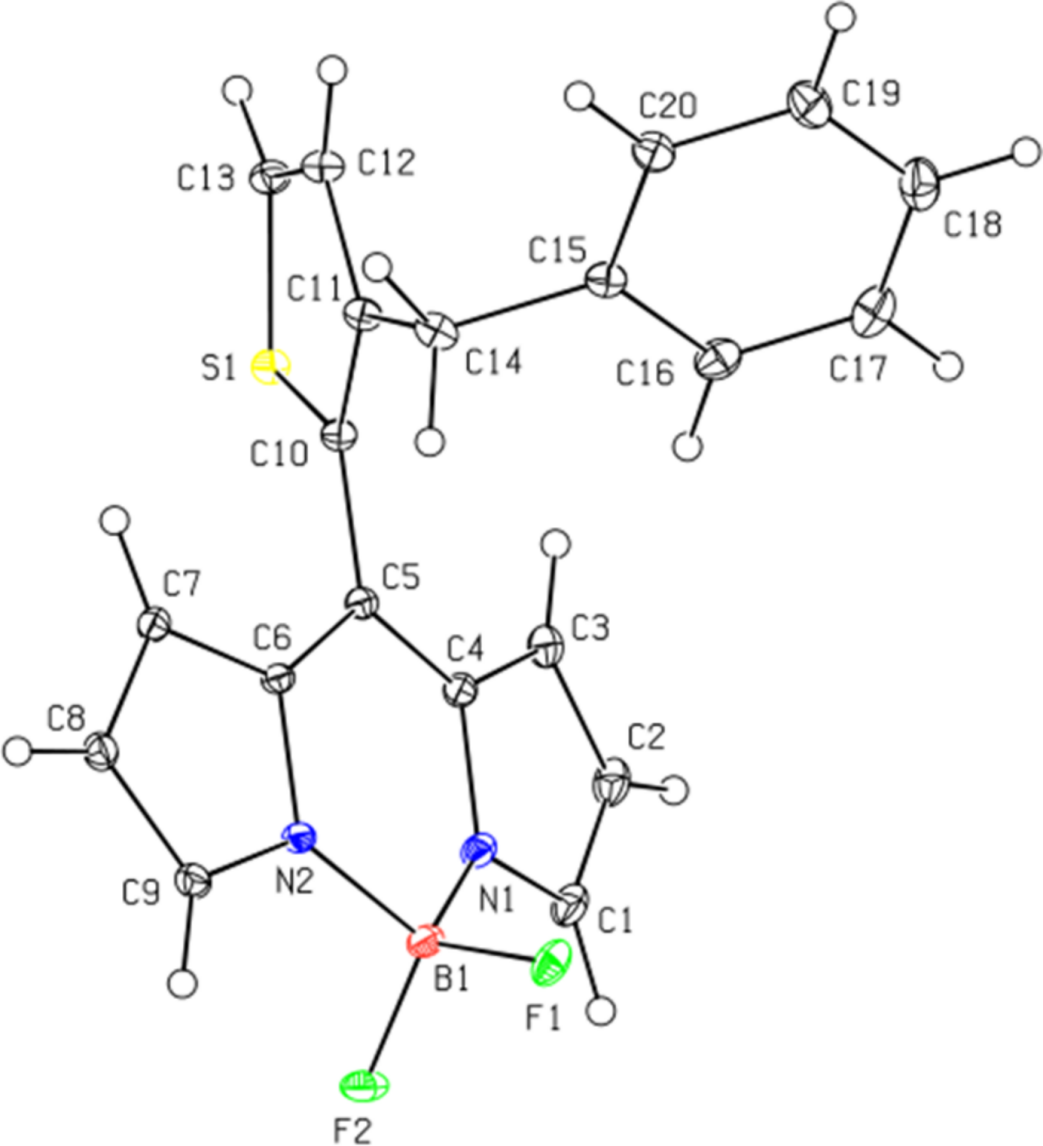
Mol­ecular structure of the title compound showing the atom labelling and ellipsoids at the 30% probability level.

In the title compound (Fig. 2 ▸), the twelve-membered ring system (B1/N1/N2/C1–C9) is essentially planar (r.m.s. deviation = 0.001 Å). The dihedral angles between the average plane of this ring and the thio­phene (S1/C10–C13) and phenyl (C15–C20) rings are 58.69 (4) and 61.41 (4)°, respectively. The thio­phene and phenyl rings subtend an angle of 81.50 (5)°. The F2—B1—F1 angle is 109.39 (8)°. The bond lengths and angles in the title compound are in good agreement with those in the compounds discussed in the *Database survey* section.

## 3. Supra­molecular features and Hirshfeld surface analysis

**Figure 3:**
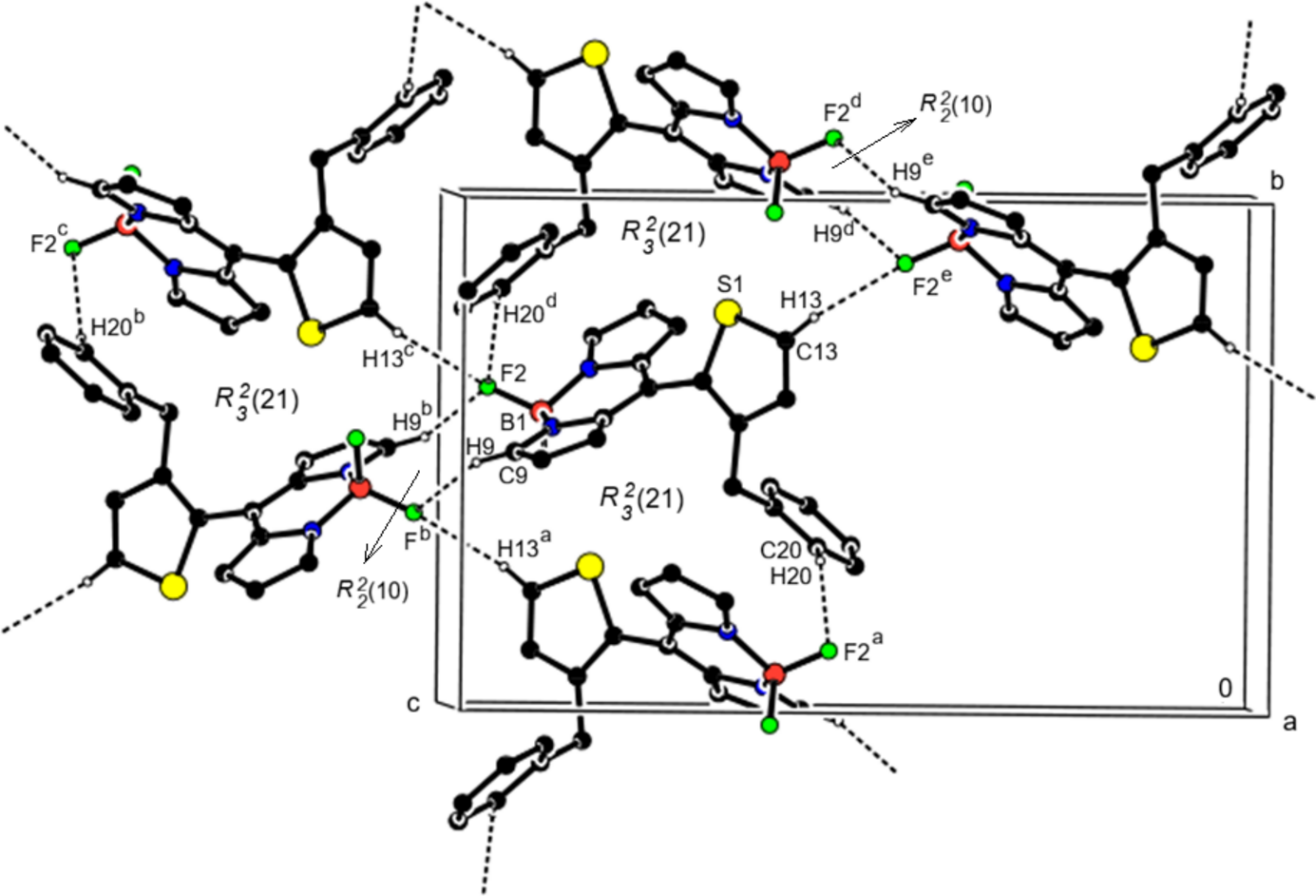
The C—H⋯F inter­actions of the title compound showing the ring motifs. Symmetry codes: (*a*) 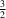 − *x*, −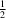 + *y*, 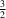 − *z*; (*b*) 1 − *x*, 1 − *y*, 2 − *z*; (*c*) −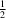 + *x*, 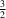 − *y*, 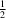 + *z*; (*d*) 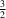 − *x*, 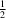 + *y*, 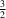 − *z*; (*e*) 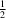 + *x*, 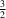 − *y*, −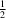 + *z*.

**Figure 6:**
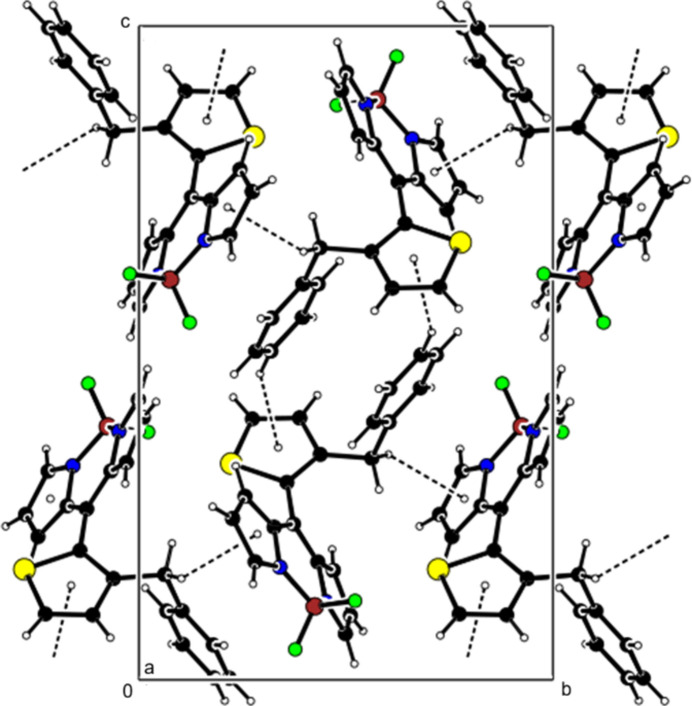
Crystal packing along the *a* axis showing C—H⋯π inter­actions (dashed lines).

In the crystal, C—H⋯F inter­actions form 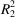
(10) and three types of 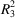
(21) ring motifs (Bernstein *et al.* 1995 ▸; Tables 1 ▸ and 2 ▸; Figs. 3 ▸, 4 ▸ and 5 ▸) around a mol­ecule, leading to the formation of layers parallel to the (101) plane. The mol­ecules are additionally connected by C—H⋯π inter­actions, forming layers parallel to the (10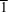) plane (Table 1 ▸; Figs. 6 ▸ and 7 ▸).

**Figure 8:**
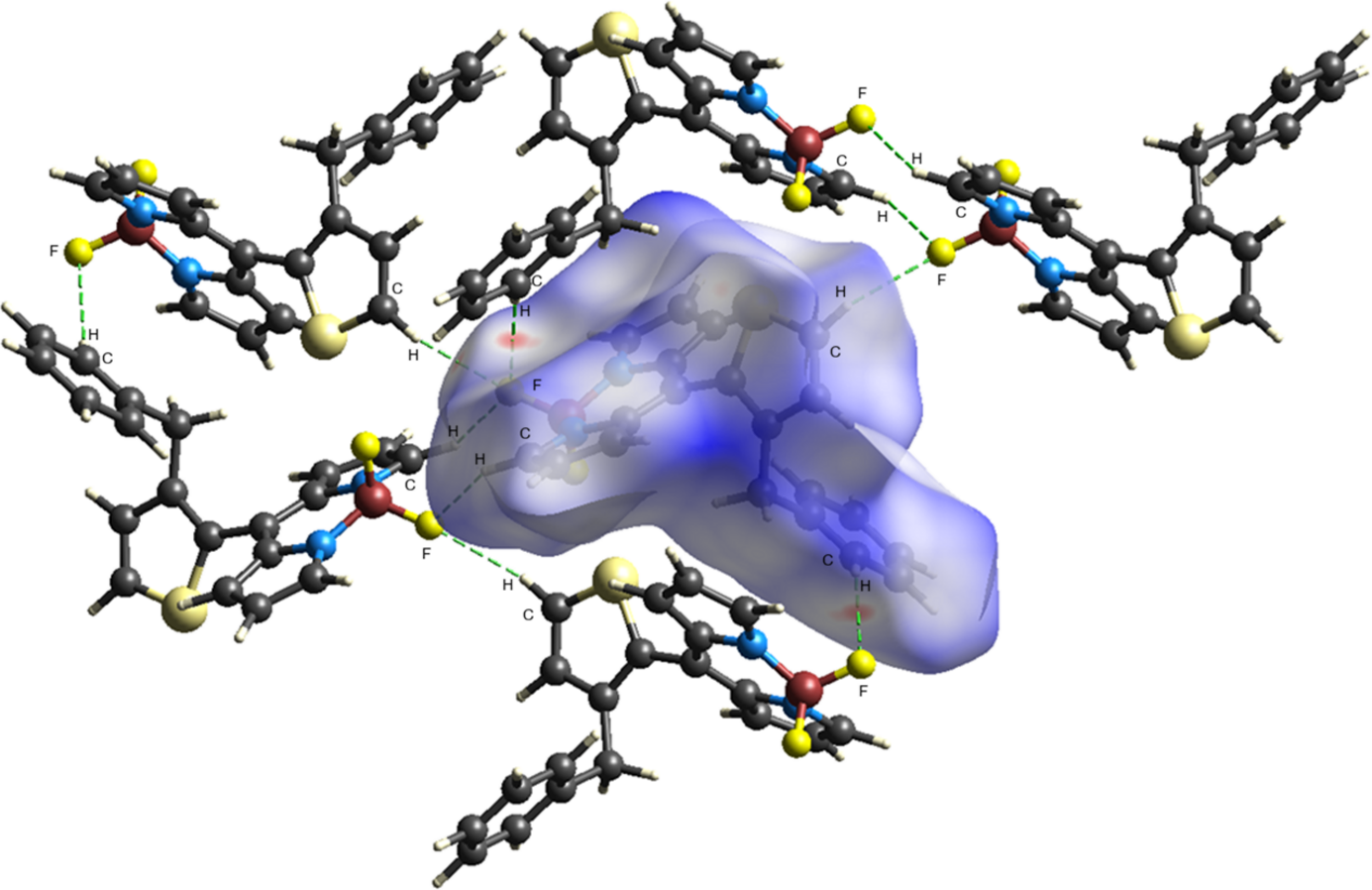
The three-dimensional Hirshfeld surface for the title compound, plotted over *d*_norm_, showing C—H⋯F inter­actions (dashed lines).

**Figure 9:**
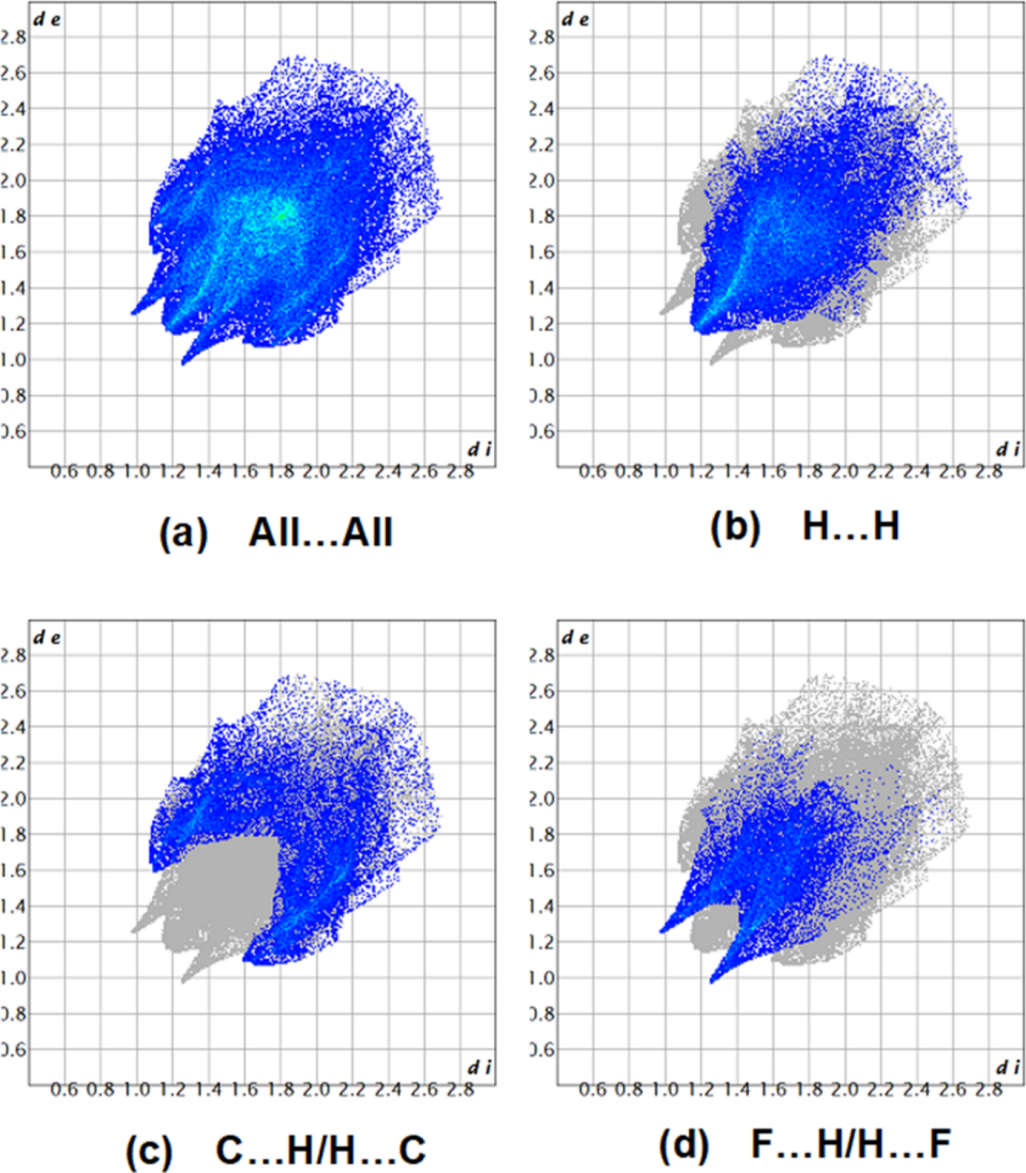
The two-dimensional fingerprint plots for the title mol­ecule showing (*a*) all inter­actions, and delineated into (*b*) H⋯H, (*c*) C⋯H/H⋯C and (*d*) F⋯H/H⋯F inter­actions. The *d*_i_ and *d*_e_ values are the closest inter­nal and external distances (in Å) from given points on the Hirshfeld surface.

A Hirshfeld surface analysis was conducted using *Crystal Explorer 17.5* (Spackman *et al.*, 2021 ▸) to view and qu­antify inter­molecular inter­actions, and to create the corresponding two-dimensional fingerprint plots. The Hirshfeld surfaces were mapped over *d*_norm_ in the range −0.2379 (red) to +1.5528 (blue) a.u. (Fig. 8 ▸). The most important inter­molecular inter­actions are the H⋯H inter­action (41.5%), which appear at the central region of the fingerprint plot with *d*_e_ = *d*_i_ ≃ 1.15 Å (Fig. 9 ▸*b*). The reciprocal C⋯H/H⋯C inter­actions appear as two symmetrical broad wings with *d*_e_ + *d*_i_ ≃ 2.75 Å and contribute 23.5% to the Hirshfeld surface (Fig. 9 ▸*c*). The reciprocal F⋯H/H⋯F inter­action with an 18.2% contribution is present as sharp symmetrical wings at diagonal axes *d*_e_ + *d*_i_ ≃ 2.2 (Fig. 9 ▸*d*). Other smaller contributions are made by S⋯H/H⋯S (6.2%), C⋯C (4.2%), N⋯H/H⋯N (2.3%), S⋯C/C⋯S (1.8%), S⋯N/N⋯S (0.9%), S⋯F/F⋯S (0.6%), N⋯C/C⋯N (0.5%) and F⋯F (0.2%) inter­actions.

## 4. Database survey

A search in the Cambridge Structural Database (CSD, version 6.00, update April 2025; Groom *et al.*, 2016 ▸) for *2,2-di­fluoro-3-aza-1-azonia-2-boranuidatri­cyclo­[7.3.0.03,7]dodeca-1(12)\,4,6,8,10-penta­ene* (twelve-membered ring moiety) gives thirteen hits, *viz.***I** (DUTLOX: Shchevnikov *et al.*, 2025 ▸), **II** (GATDIQ: Khan & Ravikanth, 2012 ▸), **III** (GATDOW: Khan & Ravikanth, 2012 ▸), **IV** (KETDAQ: Jun *et al.*, 2012*a* ▸), **V** (NARSAC: Khan *et al.*, 2012 ▸), **VI** (NARSEG: Khan *et al.*, 2012 ▸), **VII** (ROZGEU: Zhao *et al.*, 2015 ▸), **VIII** (ROZHAR: Zhao *et al.*, 2015 ▸), **IX** (ROZHEV: Zhao *et al.*, 2015 ▸), **X** (UKANUQ: Kim *et al.*, 2010 ▸), **XI** (UKANUQ01: Khan *et al.*, 2012 ▸), **XII** (ULAQOP: Sharma *et al.*, 2016 ▸) and **XIII** (XELDAV: Jun *et al.*, 2012*b* ▸).

**II**, **III**, **VII** and **VIII** crystallize in the triclinic space group *P*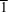. **IV** and **XII** crystallize in the ortho­rhom­bic space groups *Pbca* and *Pna*2_1_, respectively. **V**, **IX**, **X** and **XI** crystallize in the monoclinic space group *P*2_1_/*c*, while **VI**, **XIII** and **I** crystallize in the monoclinic space groups *P*2_1_/*n, C*2/*c* and *C*2/*c*, respectively.

The dihedral angle between the two ring systems (furan/thio­phene substituent and twelve-membered ring moiety) varies between 25.93 (10) and 88.13 (14)°, and is influenced by the substitution pattern and mol­ecular environment. In **I**, a thio­phene ring is affixed to the twelve-membered ring system, whilst the others are connected to a furan ring. In **I** 33.34 (6)°, in **II**, with two independent mol­ecules in the asymmetric unit, the dihedral angles are 33.31 (10) and 33.85 (9)°, in **III** 44.4 (5)°, in **IV** 88.13 (14)°, in **VIII** 84.82 (8)°, in **IX** 78.02 (9)°, in **XII** 31.24 (16)°, in **XIII** 75.60 (13)°, and in **V**, with two independent mol­ecules in the asymmetric unit, 29.4 (2) and 32.2 (2)°, respectively. In **VI**, the furan ring is disordered over two positions, the dihedral angles are 83.0 (3) and 36.9 (2)°, respectively. In **X**, with two independent mol­ecules in the asymmetric unit, the dihedral angles are 26.59 (16) and 26.92 (17)°, with similar values for **XI** [26.65 (10) and 25.93 (10)°].

In **IV**, **VII** and **IX**, C—H⋯F intra­molecular inter­actions are observed, while in **VIII** there are C—H⋯S and C—H⋯F inter­actions. In the remaining compounds, there are intra­molecular C—H⋯O hydrogen bonds involving the O atom of the furan ring. Additionally, in **VI** and **XII**, besides C—H⋯O, there are also C—H⋯F inter­actions, and in **XIII**, intra­molecular C—H⋯S inter­actions are present as well.

In compounds **I**, **II**, **III**, **V**, **VI**, **VII**, **X**, **XI**, **XII**, and **XIII**, the intra­molecular C—H⋯O inter­actions have H⋯O distances ranging from 2.29 to 2.05 Å, and C—H⋯O angles ranging from 109 to 169°. In compounds **II**, **III**, **IV**, **V**, **VI**, **VII**, **VIII**, **IX**, **X**, **XI**, **XII**, and **XIII**, the intra­molecular C—H⋯F inter­actions have H⋯F distances ranging from 2.29 to 2.55 Å, and C—H⋯F angles ranging from 110 to 179°. In compounds **VIII** and **XIII**, the intra­molecular C—H⋯S inter­actions have H⋯S distances ranging from 2.77 to 2.85 Å, and C—H⋯S angles ranging from 105 to 111°. Intra­molecular inter­actions can arise from the presence of different components attached to the main group of the mol­ecules.

## 5. Synthesis and crystallization

The BODIPY synthesis procedure was reported previously (Shchevnikov *et al.*, 2025 ▸). Dipyrrolmethane **1** (Sadikhova *et al.*, 2024 ▸) (418 mg, 1.3 mmol) was dissolved in dry DCM (20 mL), after that 2,3-di­chloro-5,6-di­cyano­benzo­quinone (DDQ, 890 mg, 3.9 mmol) was added; the reaction mixture was stirred for 30 min at r.t (TLC control), poured into water (50 mL) and extracted with DCM (3 × 30 mL). The organic layer was dried with anhydrous Na_2_SO_4_, concentrated *in vacuo* and the residue was dissolved in dry DCM (20 mL). Boron trifluoride etherate (3.3 ml, 26.3 mmol) and an equal volume of diiso­propyl­ethyl­amine (DIPEA, 3.3 ml) were added to the solution. The reaction mixture was stirred at r.t. for 1 h (TLC control) and then poured into water (50 mL), extracted with DCM (3 × 30 mL) and washed with saturated Na_2_CO_3_ (3 × 30 mL). The organic layer was dried with anhydrous Na_2_SO_4_, the target product **2** was purified by column chromatography (eluent: ethyl acetate/hexane 1:10) to give red crystals, yield 40%, 188 mg (0.52 mmol), m.p. 407–409 K. Single crystals of the title compound were grown from a mixture of ethyl acetate/hexane. IR (KBr), *ν* (cm^−1^): 1551, 1410, 1386, 1110, 1074, 828. ^1^H NMR (700.2 MHz, CDCl_3_) (*J*, Hz): *δ* 7.93 (*br.s*, 2H, H Pyr), 7.49 (*d*, *J* = 5.25 Hz, 1H, H-5 Thien), 7.21 (*t*, *J* = 7.39 Hz, 2H, H-3,5 Ph), 7.15 (*t*, *J* = 7.39 Hz, 1H, H-4 Ph), 7.01–6.98 (*m*, 5H, H Aryl + H Pyr + H-4 Thien), 6.54 (*m*, 2H, H Pyr), 3.96 (*s*, 2H, CH_2_).^13^C NMR (176.1 MHz, CDCl_3_): *δ* 167.8, 144.6 (2C), 142.9, 139.6, 139.3, 135.8, 132.5, 131.5, 130.9, 130.1, 128.8, 128.6 (2C), 128.5 (2C), 128.1, 126.4, 118.7, 35.6. ^19^F NMR (658.8 MHz, CDCl_3_): *δ* −144.8 – −145.5 (*m*, 2F). GC-MS (EI, 70 eV): *m*/*z* (%) = 364 (62) [*M*^+^], 363 (87), 343 (17), 342 (17), 288 (15), 287 (84), 286 (88), 268 (10), 267 (58), 266 (100), 172 (10).

## 6. Refinement

Crystal data, data collection and structure refinement details are summarized in Table 3 ▸. All C-bound H atoms were positioned geometrically (C—H = 0.95 and 0.99 Å) and refined using a riding model with *U*_iso_(H) = 1.2*U*_eq_(C). Owing to poor agreement between observed and calculated intensities, one outlier (0 0 2) was omitted in the final cycles of refinement.

## Supplementary Material

Crystal structure: contains datablock(s) I. DOI: 10.1107/S2056989026003889/jp2027sup1.cif

Structure factors: contains datablock(s) I. DOI: 10.1107/S2056989026003889/jp2027Isup2.hkl

CCDC reference: 2545790

Additional supporting information:  crystallographic information; 3D view; checkCIF report

## Figures and Tables

**Figure 4:**
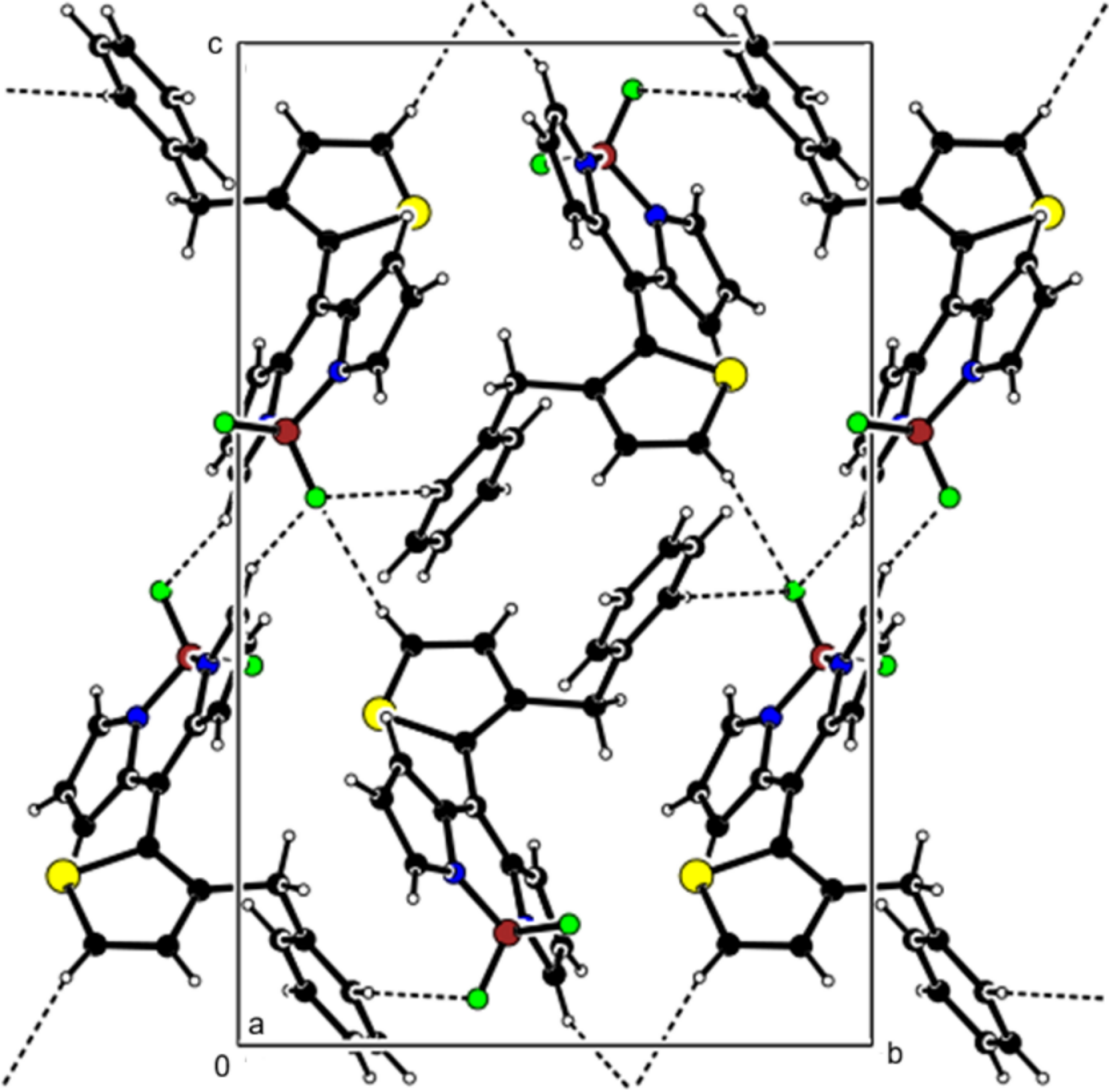
Crystal packing along the *a* axis showing C—H⋯F inter­actions (dashed lines).

**Figure 5:**
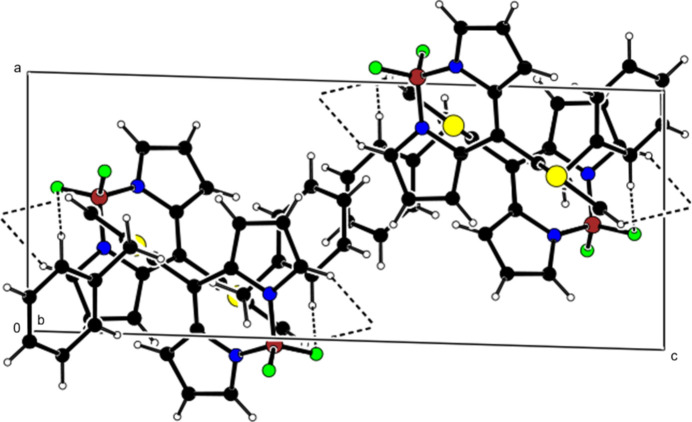
View of the C—H⋯F inter­actions in Fig. 4 ▸ along the *b* axis.

**Figure 7:**
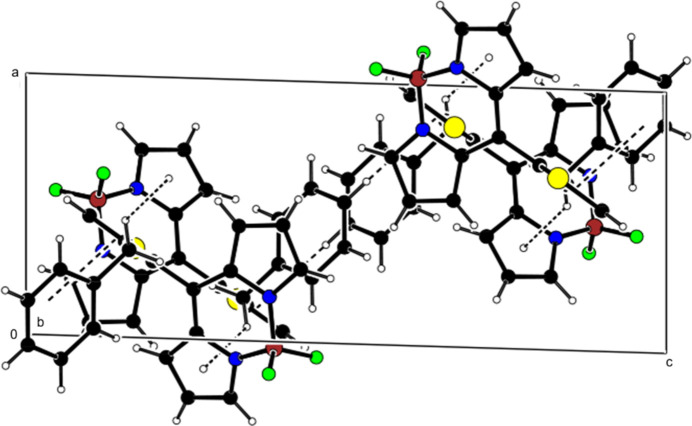
View of the C—H⋯π inter­actions in Fig. 6 ▸ along the *b* axis.

**Table 1:** Hydrogen-bond geometry (Å, °) *Cg*1 and *Cg*2 are the centroids of the S1/C10–C13 and N1/C1–C4 rings, respectively.

*D*—H⋯*A*	*D*—H	H⋯*A*	*D*⋯*A*	*D*—H⋯*A*
C9—H9⋯F2^i^	0.95	2.36	3.2017 (12)	148
C13—H13⋯F2^ii^	0.95	2.55	3.3113 (12)	137
C20—H20⋯F2^iii^	0.95	2.51	3.3221 (13)	144
C14—H14*B*⋯*Cg*2^iii^	0.99	2.81	3.5815 (11)	135
C18—H18⋯*Cg*1^iv^	0.95	2.92	3.7764 (13)	151

Symmetry codes: (i) 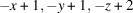; (ii) 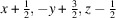; (iii) 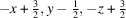; (iv) 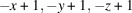.

**Table 2:** Summary of short inter­atomic contacts (Å)

Contact	Distance	Symmetry operation
H8⋯F1	2.70	1 + *x*, *y*, *z*
F2⋯H20	2.51	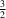 − *x*, 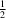 + *y*, 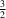 − *z*
H18⋯C13	2.87	1 − *x*, 1 − *y*, 1 − *z*
H9⋯F2	2.36	1 − *x*, 1 − *y*, 2 − *z*
F2⋯H13	2.55	− 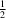 + *x*, 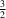 − *y*, 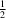 + *z*
H1⋯C17	2.80	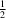 − *x*, 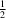 + *y*, 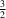 − *z*
H9⋯H8	2.52	2 − *x*, 1 − *y*, 2 − *z*
H19⋯H13	2.53	2 − *x*, 1 − *y*, 1 − *z*
H8⋯H19	2.54	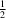 + *x*, 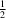 − *y*, 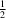 + *z*

**Table 3:** Experimental details

Crystal data	
Chemical formula	C_20_H_15_BF_2_N_2_S
*M*_r_	364.21
Crystal system, space group	Monoclinic, *P*2_1_/*n*
Temperature (K)	100
*a*, *b*, *c* (Å)	7.6652 (2), 11.9170 (2), 18.8416 (4)
β (°)	91.7200 (8)
*V* (Å^3^)	1720.33 (6)
*Z*	4
Radiation type	Mo *K*α
μ (mm^−1^)	0.21
Crystal size (mm)	0.27 × 0.15 × 0.04

Data collection	
Diffractometer	Bruker D8 QUEST PHOTON-III area detector
Absorption correction	Analytical (*SADABS*; Krause *et al.*, 2015 ▸)
*T*_min_, *T*_max_	0.702, 0.746
No. of measured, independent and observed [*I* > 2σ(*I*)] reflections	64200, 6292, 5312
*R*_int_	0.041
(sin θ/λ)_max_ (Å^−1^)	0.759

Refinement	
*R*[*F*^2^ > 2σ(*F*^2^)], *wR*(*F*^2^), *S*	0.034, 0.090, 1.03
No. of reflections	6292
No. of parameters	235
H-atom treatment	H-atom parameters constrained
Δρ_max_, Δρ_min_ (e Å^−3^)	0.45, −0.31

Computer programs: *APEX3* and *SAINT* (Bruker, 2018 ▸), *SHELXT* (Sheldrick, 2015*a* ▸), *SHELXL* (Sheldrick, 2015*b* ▸), *ORTEP-3 for Windows* (Farrugia, 2012 ▸) and *PLATON* (Spek, 2020 ▸).

## supplementary crystallographic information

### 10-(3-Benzylthiophen-2-yl)-5,5-difluoro-5H-4λ4,5λ4-dipyrrolo[1,2-c:2',1'-f][1,3,2]diazaborinine Crystal data

**Table:**

C_20_H_15_BF_2_N_2_S	*F*(000) = 752
*M**_r_* = 364.21	*D*_x_ = 1.406 Mg m^−^^3^
Monoclinic, *P*2_1_/*n*	Mo *K*α radiation, λ = 0.71073 Å
*a* = 7.6652 (2) Å	Cell parameters from 9889 reflections
*b* = 11.9170 (2) Å	θ = 2.8–32.5°
*c* = 18.8416 (4) Å	µ = 0.21 mm^−^^1^
β = 91.7200 (8)°	*T* = 100 K
*V* = 1720.33 (6) Å^3^	Plate, colourless
*Z* = 4	0.27 × 0.15 × 0.04 mm

### 10-(3-Benzylthiophen-2-yl)-5,5-difluoro-5H-4λ4,5λ4-dipyrrolo[1,2-c:2',1'-f][1,3,2]diazaborinine Data collection

**Table:**

Bruker D8 QUEST PHOTON-III area detector diffractometer	5312 reflections with *I* > 2σ(*I*)
Radiation source: fine-focus sealed X-ray tube	*R*_int_ = 0.041
φ and ω scans	θ_max_ = 32.7°, θ_min_ = 2.8°
Absorption correction: analytical (SADABS; Krause *et al.*, 2015)	*h* = −11→11
*T*_min_ = 0.702, *T*_max_ = 0.746	*k* = −17→18
64200 measured reflections	*l* = −28→28
6292 independent reflections	

### 10-(3-Benzylthiophen-2-yl)-5,5-difluoro-5H-4λ4,5λ4-dipyrrolo[1,2-c:2',1'-f][1,3,2]diazaborinine Refinement

**Table:**

Refinement on *F*^2^	Primary atom site location: difference Fourier map
Least-squares matrix: full	Secondary atom site location: difference Fourier map
*R*[*F*^2^ > 2σ(*F*^2^)] = 0.034	Hydrogen site location: inferred from neighbouring sites
*wR*(*F*^2^) = 0.090	H-atom parameters constrained
*S* = 1.03	*w* = 1/[σ^2^(*F*_o_^2^) + (0.0403*P*)^2^ + 0.7409*P*] where *P* = (*F*_o_^2^ + 2*F*_c_^2^)/3
6292 reflections	(Δ/σ)_max_ = 0.001
235 parameters	Δρ_max_ = 0.45 e Å^−^^3^
0 restraints	Δρ_min_ = −0.30 e Å^−^^3^

### 10-(3-Benzylthiophen-2-yl)-5,5-difluoro-5H-4λ4,5λ4-dipyrrolo[1,2-c:2',1'-f][1,3,2]diazaborinine Special details

**Table:**

Geometry. All esds (except the esd in the dihedral angle between two l.s. planes) are estimated using the full covariance matrix. The cell esds are taken into account individually in the estimation of esds in distances, angles and torsion angles; correlations between esds in cell parameters are only used when they are defined by crystal symmetry. An approximate (isotropic) treatment of cell esds is used for estimating esds involving l.s. planes.

### 10-(3-Benzylthiophen-2-yl)-5,5-difluoro-5H-4λ4,5λ4-dipyrrolo[1,2-c:2',1'-f][1,3,2]diazaborinine Fractional atomic coordinates and isotropic or equivalent isotropic displacement parameters (Å^2^)

**Table:**

	*x*	*y*	*z*	*U*_iso_*/*U*_eq_	
S1	0.84161 (3)	0.77669 (2)	0.66905 (2)	0.01507 (6)	
F1	0.37533 (8)	0.47800 (5)	0.87911 (4)	0.02186 (13)	
F2	0.44409 (9)	0.62246 (6)	0.95338 (3)	0.02200 (13)	
N1	0.42895 (10)	0.65968 (7)	0.82742 (4)	0.01540 (15)	
N2	0.67101 (10)	0.54855 (7)	0.88026 (4)	0.01384 (14)	
C1	0.28040 (13)	0.71956 (9)	0.81925 (6)	0.02002 (19)	
H1	0.191765	0.724323	0.853302	0.024*	
C2	0.27586 (13)	0.77380 (9)	0.75314 (6)	0.0224 (2)	
H2	0.186268	0.821940	0.735087	0.027*	
C3	0.42703 (13)	0.74382 (9)	0.71911 (6)	0.01876 (18)	
H3	0.459250	0.766234	0.672925	0.023*	
C4	0.52415 (12)	0.67355 (8)	0.76622 (5)	0.01446 (16)	
C5	0.69134 (12)	0.62665 (8)	0.76159 (5)	0.01292 (15)	
C6	0.76540 (12)	0.56795 (8)	0.81948 (5)	0.01312 (15)	
C7	0.93860 (12)	0.53056 (8)	0.83257 (5)	0.01448 (16)	
H7	1.031418	0.533229	0.800315	0.017*	
C8	0.94721 (13)	0.48929 (8)	0.90137 (5)	0.01685 (17)	
H8	1.047172	0.458677	0.925277	0.020*	
C9	0.77969 (13)	0.50136 (8)	0.92910 (5)	0.01684 (17)	
H9	0.748065	0.479417	0.975450	0.020*	
C10	0.79423 (12)	0.64260 (8)	0.69795 (5)	0.01341 (15)	
C11	0.86597 (12)	0.56204 (8)	0.65512 (5)	0.01466 (16)	
C12	0.96116 (13)	0.61169 (8)	0.59931 (5)	0.01747 (17)	
H12	1.019220	0.569191	0.564492	0.021*	
C13	0.96074 (13)	0.72614 (9)	0.60079 (5)	0.01759 (17)	
H13	1.019433	0.772085	0.567917	0.021*	
C14	0.83518 (14)	0.43748 (8)	0.66102 (5)	0.01752 (17)	
H14A	0.792936	0.420093	0.708926	0.021*	
H14B	0.946934	0.397354	0.655200	0.021*	
C15	0.70289 (13)	0.39605 (8)	0.60560 (5)	0.01605 (17)	
C16	0.52988 (14)	0.43302 (10)	0.60547 (6)	0.0230 (2)	
H16	0.494303	0.484795	0.640544	0.028*	
C17	0.40940 (15)	0.39469 (11)	0.55446 (7)	0.0277 (2)	
H17	0.292221	0.420597	0.554866	0.033*	
C18	0.45901 (16)	0.31889 (11)	0.50298 (7)	0.0277 (2)	
H18	0.376319	0.292630	0.468269	0.033*	
C19	0.63037 (16)	0.28183 (10)	0.50265 (6)	0.0261 (2)	
H19	0.665301	0.229907	0.467558	0.031*	
C20	0.75163 (14)	0.32035 (9)	0.55354 (6)	0.01976 (18)	
H20	0.868880	0.294677	0.552698	0.024*	
B1	0.47484 (14)	0.57533 (9)	0.88759 (6)	0.01587 (18)	

### 10-(3-Benzylthiophen-2-yl)-5,5-difluoro-5H-4λ4,5λ4-dipyrrolo[1,2-c:2',1'-f][1,3,2]diazaborinine Atomic displacement parameters (Å^2^)

**Table:**

	*U*^11^	*U*^22^	*U*^33^	*U*^12^	*U*^13^	*U*^23^
S1	0.01764 (10)	0.01170 (10)	0.01596 (10)	−0.00004 (7)	0.00183 (7)	0.00133 (7)
F1	0.0164 (3)	0.0159 (3)	0.0335 (3)	−0.0049 (2)	0.0042 (2)	−0.0008 (2)
F2	0.0260 (3)	0.0227 (3)	0.0179 (3)	−0.0011 (2)	0.0099 (2)	−0.0029 (2)
N1	0.0126 (3)	0.0151 (3)	0.0186 (4)	−0.0003 (3)	0.0032 (3)	−0.0024 (3)
N2	0.0145 (3)	0.0149 (3)	0.0123 (3)	−0.0024 (3)	0.0025 (3)	−0.0006 (3)
C1	0.0131 (4)	0.0194 (4)	0.0277 (5)	0.0001 (3)	0.0023 (3)	−0.0045 (4)
C2	0.0142 (4)	0.0228 (5)	0.0298 (5)	0.0031 (4)	−0.0031 (4)	−0.0019 (4)
C3	0.0168 (4)	0.0191 (4)	0.0202 (4)	0.0011 (3)	−0.0031 (3)	0.0002 (3)
C4	0.0137 (4)	0.0140 (4)	0.0157 (4)	−0.0001 (3)	0.0010 (3)	−0.0019 (3)
C5	0.0141 (4)	0.0115 (4)	0.0133 (4)	−0.0009 (3)	0.0015 (3)	−0.0012 (3)
C6	0.0133 (3)	0.0137 (4)	0.0125 (3)	−0.0014 (3)	0.0026 (3)	−0.0003 (3)
C7	0.0131 (4)	0.0147 (4)	0.0157 (4)	−0.0009 (3)	0.0006 (3)	−0.0003 (3)
C8	0.0164 (4)	0.0184 (4)	0.0156 (4)	−0.0013 (3)	−0.0020 (3)	0.0010 (3)
C9	0.0188 (4)	0.0185 (4)	0.0132 (4)	−0.0035 (3)	0.0004 (3)	0.0006 (3)
C10	0.0152 (4)	0.0121 (4)	0.0130 (4)	0.0003 (3)	0.0013 (3)	0.0015 (3)
C11	0.0175 (4)	0.0135 (4)	0.0131 (4)	0.0021 (3)	0.0013 (3)	0.0010 (3)
C12	0.0200 (4)	0.0180 (4)	0.0146 (4)	0.0024 (3)	0.0042 (3)	0.0010 (3)
C13	0.0186 (4)	0.0188 (4)	0.0155 (4)	−0.0004 (3)	0.0032 (3)	0.0030 (3)
C14	0.0251 (5)	0.0124 (4)	0.0151 (4)	0.0029 (3)	0.0007 (3)	0.0004 (3)
C15	0.0208 (4)	0.0118 (4)	0.0157 (4)	0.0006 (3)	0.0027 (3)	0.0021 (3)
C16	0.0213 (5)	0.0207 (5)	0.0274 (5)	0.0029 (4)	0.0057 (4)	−0.0007 (4)
C17	0.0184 (5)	0.0273 (5)	0.0373 (6)	−0.0005 (4)	0.0006 (4)	0.0043 (5)
C18	0.0267 (5)	0.0263 (5)	0.0295 (5)	−0.0070 (4)	−0.0058 (4)	0.0023 (4)
C19	0.0308 (5)	0.0226 (5)	0.0248 (5)	−0.0022 (4)	−0.0013 (4)	−0.0066 (4)
C20	0.0225 (4)	0.0164 (4)	0.0204 (4)	0.0015 (3)	0.0014 (4)	−0.0026 (3)
B1	0.0155 (4)	0.0151 (4)	0.0173 (4)	−0.0025 (3)	0.0049 (3)	−0.0021 (3)

### 10-(3-Benzylthiophen-2-yl)-5,5-difluoro-5H-4λ4,5λ4-dipyrrolo[1,2-c:2',1'-f][1,3,2]diazaborinine Geometric parameters (Å, º)

**Table:**

S1—C13	1.7093 (10)	C8—H8	0.9500
S1—C10	1.7302 (9)	C9—H9	0.9500
F1—B1	1.3948 (12)	C10—C11	1.3792 (13)
F2—B1	1.3875 (12)	C11—C12	1.4263 (13)
N1—C1	1.3488 (13)	C11—C14	1.5076 (14)
N1—C4	1.3928 (12)	C12—C13	1.3641 (14)
N1—B1	1.5477 (14)	C12—H12	0.9500
N2—C9	1.3457 (12)	C13—H13	0.9500
N2—C6	1.3921 (11)	C14—C15	1.5161 (14)
N2—B1	1.5474 (13)	C14—H14A	0.9900
C1—C2	1.4028 (16)	C14—H14B	0.9900
C1—H1	0.9500	C15—C20	1.3920 (14)
C2—C3	1.3878 (14)	C15—C16	1.3974 (15)
C2—H2	0.9500	C16—C17	1.3900 (17)
C3—C4	1.4155 (14)	C16—H16	0.9500
C3—H3	0.9500	C17—C18	1.3869 (18)
C4—C5	1.4033 (13)	C17—H17	0.9500
C5—C6	1.4015 (13)	C18—C19	1.3860 (18)
C5—C10	1.4672 (12)	C18—H18	0.9500
C6—C7	1.4151 (13)	C19—C20	1.3929 (15)
C7—C8	1.3861 (13)	C19—H19	0.9500
C7—H7	0.9500	C20—H20	0.9500
C8—C9	1.4081 (14)		
			
C13—S1—C10	91.91 (5)	C10—C11—C14	125.18 (9)
C1—N1—C4	107.71 (9)	C12—C11—C14	123.20 (8)
C1—N1—B1	126.85 (8)	C13—C12—C11	113.47 (9)
C4—N1—B1	124.78 (8)	C13—C12—H12	123.3
C9—N2—C6	107.79 (8)	C11—C12—H12	123.3
C9—N2—B1	127.48 (8)	C12—C13—S1	111.67 (7)
C6—N2—B1	124.72 (8)	C12—C13—H13	124.2
N1—C1—C2	110.08 (9)	S1—C13—H13	124.2
N1—C1—H1	125.0	C11—C14—C15	111.89 (8)
C2—C1—H1	125.0	C11—C14—H14A	109.2
C3—C2—C1	107.03 (9)	C15—C14—H14A	109.2
C3—C2—H2	126.5	C11—C14—H14B	109.2
C1—C2—H2	126.5	C15—C14—H14B	109.2
C2—C3—C4	107.05 (9)	H14A—C14—H14B	107.9
C2—C3—H3	126.5	C20—C15—C16	118.54 (10)
C4—C3—H3	126.5	C20—C15—C14	120.55 (9)
N1—C4—C5	120.42 (8)	C16—C15—C14	120.90 (9)
N1—C4—C3	108.10 (8)	C17—C16—C15	120.56 (10)
C5—C4—C3	131.36 (9)	C17—C16—H16	119.7
C6—C5—C4	119.99 (8)	C15—C16—H16	119.7
C6—C5—C10	119.05 (8)	C18—C17—C16	120.48 (11)
C4—C5—C10	120.90 (8)	C18—C17—H17	119.8
N2—C6—C5	120.98 (8)	C16—C17—H17	119.8
N2—C6—C7	108.20 (8)	C19—C18—C17	119.37 (11)
C5—C6—C7	130.45 (8)	C19—C18—H18	120.3
C8—C7—C6	107.02 (8)	C17—C18—H18	120.3
C8—C7—H7	126.5	C18—C19—C20	120.32 (11)
C6—C7—H7	126.5	C18—C19—H19	119.8
C7—C8—C9	107.01 (8)	C20—C19—H19	119.8
C7—C8—H8	126.5	C15—C20—C19	120.73 (10)
C9—C8—H8	126.5	C15—C20—H20	119.6
N2—C9—C8	109.97 (8)	C19—C20—H20	119.6
N2—C9—H9	125.0	F2—B1—F1	109.39 (8)
C8—C9—H9	125.0	F2—B1—N2	110.78 (8)
C11—C10—C5	128.43 (8)	F1—B1—N2	110.35 (8)
C11—C10—S1	111.57 (7)	F2—B1—N1	110.53 (8)
C5—C10—S1	119.99 (7)	F1—B1—N1	110.16 (8)
C10—C11—C12	111.37 (8)	N2—B1—N1	105.59 (7)
			
C4—N1—C1—C2	−0.25 (12)	C13—S1—C10—C5	178.07 (8)
B1—N1—C1—C2	−171.22 (9)	C5—C10—C11—C12	−178.38 (9)
N1—C1—C2—C3	1.12 (12)	S1—C10—C11—C12	0.69 (11)
C1—C2—C3—C4	−1.50 (12)	C5—C10—C11—C14	7.25 (16)
C1—N1—C4—C5	175.67 (9)	S1—C10—C11—C14	−173.69 (8)
B1—N1—C4—C5	−13.13 (14)	C10—C11—C12—C13	0.24 (13)
C1—N1—C4—C3	−0.70 (11)	C14—C11—C12—C13	174.74 (9)
B1—N1—C4—C3	170.50 (9)	C11—C12—C13—S1	−1.06 (12)
C2—C3—C4—N1	1.38 (11)	C10—S1—C13—C12	1.22 (8)
C2—C3—C4—C5	−174.45 (10)	C10—C11—C14—C15	100.99 (11)
N1—C4—C5—C6	−1.70 (14)	C12—C11—C14—C15	−72.74 (12)
C3—C4—C5—C6	173.70 (10)	C11—C14—C15—C20	116.03 (10)
N1—C4—C5—C10	−178.78 (8)	C11—C14—C15—C16	−63.81 (12)
C3—C4—C5—C10	−3.38 (16)	C20—C15—C16—C17	0.12 (16)
C9—N2—C6—C5	−173.72 (9)	C14—C15—C16—C17	179.97 (10)
B1—N2—C6—C5	7.51 (14)	C15—C16—C17—C18	0.16 (18)
C9—N2—C6—C7	0.03 (10)	C16—C17—C18—C19	−0.21 (19)
B1—N2—C6—C7	−178.74 (8)	C17—C18—C19—C20	−0.01 (18)
C4—C5—C6—N2	4.41 (14)	C16—C15—C20—C19	−0.34 (16)
C10—C5—C6—N2	−178.46 (8)	C14—C15—C20—C19	179.81 (10)
C4—C5—C6—C7	−167.78 (9)	C18—C19—C20—C15	0.29 (18)
C10—C5—C6—C7	9.35 (15)	C9—N2—B1—F2	43.37 (13)
N2—C6—C7—C8	−0.24 (11)	C6—N2—B1—F2	−138.11 (9)
C5—C6—C7—C8	172.72 (10)	C9—N2—B1—F1	−77.93 (12)
C6—C7—C8—C9	0.34 (11)	C6—N2—B1—F1	100.59 (10)
C6—N2—C9—C8	0.19 (11)	C9—N2—B1—N1	163.05 (9)
B1—N2—C9—C8	178.91 (9)	C6—N2—B1—N1	−18.42 (12)
C7—C8—C9—N2	−0.34 (11)	C1—N1—B1—F2	−49.37 (13)
C6—C5—C10—C11	58.94 (14)	C4—N1—B1—F2	141.13 (9)
C4—C5—C10—C11	−123.95 (11)	C1—N1—B1—F1	71.64 (12)
C6—C5—C10—S1	−120.06 (8)	C4—N1—B1—F1	−97.87 (10)
C4—C5—C10—S1	57.05 (11)	C1—N1—B1—N2	−169.22 (9)
C13—S1—C10—C11	−1.09 (8)	C4—N1—B1—N2	21.27 (12)

### 10-(3-Benzylthiophen-2-yl)-5,5-difluoro-5H-4λ4,5λ4-dipyrrolo[1,2-c:2',1'-f][1,3,2]diazaborinine Hydrogen-bond geometry (Å, º)

*Cg*1 and *Cg*2 are the centroids of the S1/C10–C13 and N1/C1–C4 rings, respectively.

**Table:**

*D*—H···*A*	*D*—H	H···*A*	*D*···*A*	*D*—H···*A*
C9—H9···F2^i^	0.95	2.36	3.2017 (12)	148
C13—H13···F2^ii^	0.95	2.55	3.3113 (12)	137
C20—H20···F2^iii^	0.95	2.51	3.3221 (13)	144
C14—H14*B*···*Cg*2^iii^	0.99	2.81	3.5815 (11)	135
C18—H18···*Cg*1^iv^	0.95	2.92	3.7764 (13)	151

Symmetry codes: (i) −*x*+1, −*y*+1, −*z*+2; (ii) *x*+1/2, −*y*+3/2, *z*−1/2; (iii) −*x*+3/2, *y*−1/2, −*z*+3/2; (iv) −*x*+1, −*y*+1, −*z*+1.

## References

[bb1] Bernstein, J., Davis, R. E., Shimoni, L. & Chang, N.-L. (1995). *Angew. Chem. Int. Ed. Engl.* **34**, 1555–1573.

[bb2] Boens, N., Verbelen, B., Ortiz, M. J., Jiao, L. & Dehaen, W. (2019). *Coord. Chem. Rev.* **399**, 213024.

[bb3] Bruker (2018). *APEX3* and *SAINT*. Bruker AXS Inc., Madison, Wisconsin, USA.

[bb4] Farrugia, L. J. (2012). *J. Appl. Cryst.* **45**, 849–854.

[bb5] Groom, C. R., Bruno, I. J., Lightfoot, M. P. & Ward, S. C. (2016). *Acta Cryst.* B**72**, 171–179. 10.1107/S2052520616003954 PMC4822653 27048719

[bb6] Jun, T., Kim, K., Lee, K. M., Benniston, A. C. & Churchill, D. G. (2012*a*). *J. Coord. Chem.* **65**, 4299–4314.

[bb7] Jun, T., Kim, K., Lee, K. M., Murale, D. P., Singh, A. P., Natsagdorj, A., Liew, H., Suh, Y.-H. & Churchill, D. G. (2012*b*). *J. Porphyrins Phthalocyanines* **16**, 1201–1208.

[bb8] Khan, T. K. & Ravikanth, M. (2012). *Tetrahedron* **68**, 830–840.

[bb9] Kim, K., Jo, C., Easwaramoorthi, S., Sung, J., Kim, D. H. & Churchill, D. G. (2010). *Inorg. Chem.* **49**, 4881–4894. 10.1021/ic902467h 20420417

[bb10] Krause, L., Herbst-Irmer, R., Sheldrick, G. M. & Stalke, D. (2015). *J. Appl. Cryst.* **48**, 3–10. 10.1107/S1600576714022985 PMC4453166 26089746

[bb11] Lincoln, R., Greene, L. E., Krumova, K., Ding, Z. & Cosa, G. (2014). *J. Phys. Chem. A* **118**, 10622–10630. 10.1021/jp5059148 25066755

[bb12] Loudet, A. & Burgess, K. (2007). *Chem. Rev.* **107**, 4891–4932. 10.1021/cr078381n 17924696

[bb13] Lu, H., Mack, J., Yang, Y. & Shen, Z. (2014). *Chem. Soc. Rev.* **43**, 4778–4823. 10.1039/c4cs00030g 24733589

[bb14] Martynov, V. I. & Pakhomov, A. A. (2021). *Russ. Chem. Rev.* **90**, 1213–1262.

[bb15] Ni, Y. & Wu, J. (2014). *Org. Biomol. Chem.* **12**, 3774–3791. 10.1039/c3ob42554a 24781214

[bb16] Ozdemir, T., Kostereli, Z., Guliyev, R., Yalcin, S., Dede, Y. & Akkaya, E. U. (2014). *RSC Adv.* **4**, 14915–14918.

[bb17] Poddar, M. & Misra, R. (2020). *Coord. Chem. Rev.* **421**, 213462.

[bb18] Sadikhova, N. D., Atioğlu, Z., Guliyeva, N. A., Shelukho, E. R., Polyanskaya, D. K., Khrustalev, V. N. & Bhattarai, A. (2024). *Structure Reports* **80**, 72–77. 10.1107/S2056989023010800 PMC10833363 38312153

[bb19] Sharma, R., Lakshmi, V., Chatterjee, T. & Ravikanth, M. (2016). *New J. Chem.* **40**, 5855–5860.

[bb20] Shchevnikov, D. M., Kutasevich, A. G., Khrustalev, V. N., Guliyeva, N. A., Hasanov, K. I., Akkurt, M. & Manahelohe, G. M. (2025). *Acta Cryst.* E**81**, 582–586. 10.1107/S2056989025004888 PMC12230615 40630656

[bb21] Sheldrick, G. M. (2015*a*). *Acta Cryst.* A**71**, 3–8.

[bb22] Sheldrick, G. M. (2015*b*). *Acta Cryst.* C**71**, 3–8.

[bb23] Spackman, P. R., Turner, M. J., McKinnon, J. J., Wolff, S. K., Grimwood, D. J., Jayatilaka, D. & Spackman, M. A. (2021). *J. Appl. Cryst.* **54**, 1006–1011. 10.1107/S1600576721002910 PMC8202033 34188619

[bb24] Spector, D. V., Abramchuk, D. S., Bykusov, V. V., Zharova, A. O., Egorova, E. S., Voskresenskaya, A. S., Olovyanishnikov, A. R., Kuzmichev, I. A., Bubley, A. A., Antipin, R. L., Beloglazkina, E. K. & Krasnovskaya, O. O. (2024). *Russ. Chem. Rev.* **93**, RCR5136.

[bb25] Spek, A. L. (2020). *Acta Cryst.* E**76**, 1–11. 10.1107/S2056989019016244 PMC6944088 31921444

[bb26] Ulrich, G., Ziessel, R. & Harriman, A. (2008). *Angew. Chem. Int. Ed.* **47**, 1184–1201. 10.1002/anie.200702070 18092309

[bb27] Waly, S. M., Karlsson, J. K. G., Waddell, P. G., Benniston, A. C. & Harriman, A. (2022). *J. Phys. Chem. A* **126**, 1530–1541. 10.1021/acs.jpca.2c00035 PMC9097531 35230124

[bb28] Wang, D., Wang, X., Zhou, S., Gu, P., Zhu, X., Wang, C. & Zhang, Q. (2023). *Coord. Chem. Rev.* **482**, 215074.

[bb29] Zhao, N., Vicente, M. G. H., Fronczek, F. R. & Smith, K. M. (2015). *Chem. Eur. J.* **21**, 6181–6192. 10.1002/chem.201406550 PMC4382426 25761150

